# Binder Jetting Additive Manufacturing of High Porosity 316L Stainless Steel Metal Foams

**DOI:** 10.3390/ma13173744

**Published:** 2020-08-24

**Authors:** Ganesh Kumar Meenashisundaram, Zhengkai Xu, Mui Ling Sharon Nai, Shenglu Lu, Jyi Sheuan Ten, Jun Wei

**Affiliations:** Metal and Ceramic Forming Group, Singapore institute of Manufacturing Technology, 73 Nanyang Drive, Singapore 637662, Singapore; mganesh@simtech.a-star.edu.sg (G.K.M.); xu_zhengkai@simtech.a-star.edu.sg (Z.X.); peter-1014@hotmail.com (S.L.); ten_jyi_sheuan@simtech.a-star.edu.sg (J.S.T.); junwei@hit.edu.cn (J.W.)

**Keywords:** 316L stainless steel, high porosity, pore openness index, pore size, binder saturation rate, isothermal sintering temperatures, Young’s modulus, compression properties

## Abstract

High porosity (40% to 60%) 316L stainless steel containing well-interconnected open-cell porous structures with pore openness index of 0.87 to 1 were successfully fabricated by binder jetting and subsequent sintering processes coupled with a powder space holder technique. Mono-sized (30 µm) and 30% (by volume) spherically shaped poly(methyl methacrylate) (PMMA) powder was used as the space holder material. The effects of processing conditions such as: (1) binder saturation rates (55%, 100% and 150%), and (2) isothermal sintering temperatures (1000 °C to 1200 °C) on the porosity of 316L stainless steel parts were studied. By varying the processing conditions, porosity of 40% to 45% were achieved. To further increase the porosity values of 316L stainless steel parts, 30 vol. % (or 6 wt. %) of PMMA space holder particles were added to the 3D printing feedstock and porosity values of 57% to 61% were achieved. Mercury porosimetry results indicated pore sizes less than 40 µm for all the binder jetting processed 316L stainless steel parts. Anisotropy in linear shrinkage after the sintering process was observed for the SS316L parts with the largest linear shrinkage in the Z direction. The Young’s modulus and compression properties of 316L stainless steel parts decreased with increasing porosity and low Young’s modulus values in the range of 2 GPa to 29 GPa were able to be achieved. The parts fabricated by using pure 316L stainless steel feedstock sintered at 1200 °C with porosity of ~40% exhibited the maximum overall compressive properties with 0.2% compressive yield strength of 52.7 MPa, ultimate compressive strength of 520 MPa, fracture strain of 36.4%, and energy absorption of 116.7 MJ/m^3^, respectively. The Young’s modulus and compression properties of the binder jetting processed 316L stainless steel parts were found to be on par with that of the conventionally processed porous 316L stainless steel parts and even surpassed those having similar porosities, and matched to that of the cancellous bone types.

## 1. Introduction

316L stainless steel (SS316L), a quotidian austenitic steel, offers a wide range of applications in the marine, energy, aerospace, semiconductor and medical industries due to its high strength and corrosion resistance [1]. High porosity metal parts may exhibit excellent properties such as low density, high strength-to-weight ratio, high gas and liquid permeability, high thermal conductivity and excellent energy absorption properties [2]. Low modulus biomaterials with high porosity and open-cell porous structures are of particular interests targeting orthopedic implant applications favoring bone in-growth [3]. SS316L is one of the most commonly used biomaterials for orthopedic implant applications due to its outstanding mechanical properties and bio-corrosion resistance, considerable biocompatibility, and cheaper price when compared to titanium [4]. Also, high corrosion resistant and sintered porous SS316L parts containing open-cell porous structures are the most preferred materials for filtration applications where resistance to high pressures and temperatures are essential especially in the presence of oxidizing acids or high chlorides [5].

Conventional processes can be used for fabricating porous metal parts with open and closed cell porous structures. The simplest liquid-state process for fabricating closed-cell porous metal parts is by adding foaming agent or injecting inert gas to the melts which are later cooled-down in a casting process. Investment casting with open-cell polymer as a preform can also be used to fabricate porous metal parts. After the liquid metal is injected and solidified, the polymer is burnt off resulting in an open-cell porous metal. Liquid-state processes are more applicable for low melting point metals such as aluminium [6]. The porous metal parts fabricated by using the liquid-state processes usually exhibit relatively larger and irregular pores [6]. Solid-state processes such as powder metallurgy technique can also be used to fabricate porous metal parts at much lower processing temperatures with: (a) low compaction pressure or loose powders sintered at lower temperatures, and (b) by using powder space holder technique (PSH) [7]. Solid-state processes are more suitable for high melting point metals such as steels. Some of the most commonly used powder space holder materials are: (1) carbamide [8], (2) ammonium bicarbonate [9], (3) poly(methyl methacrylate) or PMMA [10] and (4) sodium chloride [11]. The powder shape and volume of space holder materials added to the feedstock together with the sintering conditions will determine the pore characteristics (pore size and volume) formed within the metal parts. However, the conventional powder metallurgy technique coupled with PSH exhibit poor process repeatability [12]. Further, it is difficult to achieve uniform pore sizes throughout the 3D part [12]. Near-net-shaped porous metallic components with complex geometric features and micron-sized well-interconnected pores with uniform pore geometry cannot be easily fabricated by using the conventional powder metallurgy technique due to the sophisticated compaction process requiring complex and expensive tooling specific to the part shape.

Conventional solid-state processes such as metal injection moulding (MIM) coupled with PSH technique combines the advantage of plastic injection moulding and powder metallurgy techniques such as material versatility, fabrication of complex geometries and small parts with tight tolerances. MIM process consists of four consecutive processing steps: (1) feedstock preparation by mixing of powders and binder materials, (2) injection moulding or high-pressure injection of feedstock into a mould, (3) debinding or process of removing binder materials, and (4) sintering process which is usually conducted at protective atmospheres or vacuum at a temperature well below the melting point of metal. Some of the common defects in MIM parts such as blistering, cracking, incomplete fill, etc., can generate directly after the moulding process or would manifest during subsequent processing steps. Further, debinding and adequate sintering play a vital role to effectively fabricate porous metal parts with high mechanical properties without contamination and demands experimental verification. Recently, Xie et al. [13] and Gulsoy et al. [10] studied the thermal decomposition of PMMA and the best isothermal sintering temperatures for MIM-processed SS316L with feedstock mixed with PMMA as powder space holder (PSH) to achieve the desired pore features and mechanical properties, respectively. The results indicated that PMMA particles of 10 µm to 41 µm size can be used as space holder materials to fabricate microporous metal parts. In the case of SS316L stainless steel containing PMMA as PSH, strong neck connections between SS316L powders during sintering was found to occur at isothermal sintering temperature of 1200 °C and was clearly observed through microstructural investigations in porous SS316L parts fabricated by using 40 to 60 vol. % PMMA [10]. Open-cell stainless steel foams were also fabricated by impregnation of stainless steel slurry into polymer foam followed by sintering [14,15]. Hemant et al. [15] fabricated high porosity (68% to 81%) open-cell austenitic stainless steel parts by impregnation of stainless steel slurry into polyurethane foam followed by sintering at 1200 °C for 1h and investigated their microstructure and mechanical properties. The results indicated that the mechanical properties improve with increase in the relative density of the parts and compressive yield stress, elastic modulus and energy absorption properties were in the range of 5.2–10.5 MPa, 2.01–7.03 GPa, and 1.2–3.5 MJ/m^3^. The impregnation methods are simple however achieving precise control of pore size with interconnected pores and improved mechanical properties is a real challenge [15].

Due to the recent advancements in the field of metal additive manufacturing (AM), several processes such as: (a) Selective Laser Melting (SLM) [16], (b) Electron Beam Melting (EBM) [17,18], (c) Selective Laser Sintering (SLS) [19], (d) Direct Metal Laser Deposition (DMLD) [20] and (e) Ink jet 3D printing and binder jetting have been extensively explored to fabricate high porosity functional metal parts with desired pore characteristics. Among these AM processes, SLM, EBM, SLS and DMLD are energy-based processes that use high energy laser/electron beam to melt or sinter the metal powders layer-by-layer to form the 3D parts. They are more commonly used to fabricate porous metal parts with complex shapes directly from the digital CAD models by using pores-by-design approach. But, in the case of micro-porous open-cell porous structures, designing and fabricating such fine micro-pores throughout the 3D part are still in the very initial stage of research. They are difficult to achieve through pores-by-design approach due to the following reasons: (1) software limitations to design such micro-pore features throughout the 3D part, and (2) during processing, there are high chances for the loose metal powders to get trapped within the micro-pores and subsequent processing at high temperatures makes the powder removal difficult. Achieving open-cell porous structures through pores-by-processing route via the energy-based AM processes is under very initial stage of research and it is challenging to achieve the desired pore size and volume of geometrically undefined pores generated by energy-based AM processing [21].

In the additive manufacturing community, binder jetting is renowned for easy fabrication of porous parts with open-cell porous structures by using pores-by-processing approach. Contrary to the powder bed fusion energy-based technologies, binder jetting operates at ambient environment and requires no support structures. Binder jetting consists of four consecutive process steps: (1) preparation of powder bed with a spread of fine layer of powders, (2) 3D printing of part by successively adding material layer-by-layer and selectively dispensing binders from the print head every layer as per the part’s cross section, (3) as-printed parts are cured at low temperatures (typically up to 200 °C for 12 h based on the binders used; usually solvent or aqueous based for metals), (4) finally, the parts are debinded and sintered similar to the MIM process. Binder jetting provides a freeform fabrication solution for creating complex shaped porous metal structures that are difficult to be fabricated using the conventional processes such as MIM without the need for expensive moulds and tools. A brief literature review of the works on the fabrication of porous metal parts with binder jetting is discussed in the Table 1. The literature search results indicate that the porous binder jetting parts are focused mostly for biomedical applications. The binder jetting original equipment manufacturers and researchers have recently found the applications for the binder jetting manufactured porous parts for the fabrication of high efficiency metal filters for air purification and protection in response to the current COVID-19 crisis. ExOne and the University of Pittsburgh reported their research in developing porous copper parts for antimicrobial filtration applications for use in the reusable and serializable respirators [22].

Due to the advantages of binder jetting technology aiding the fabrication of porous parts, in the present work, it was chosen as the additive manufacturing technique to fabricate porous SS316L parts. To further increase the porosity of binder jetting processed parts, an appropriate powder space holder material (PSH) should be added to the feedstock. Accordingly, in the present study, 30 vol. % (or 6 wt. %) PMMA with spherical morphology and size of 30 µm is proposed as the PSH material. The volume fraction of PMMA (30 vol. %) was chosen to be less than the total volume of ink containing binders utilized during the binder jetting process. The powder bed packing density can range between the apparent and tapped density of the powders. Considering ink and binders during binder jetting penetrate and fill the interstitial void spaces between the powder particles, the volume of PMMA in the feedstock was chosen to be less than the total volume of ink and binders used during binder jetting. Or else, during debinding and sintering, the coordination number between the SS316L powder particles will be very low which will affect the binder jet part integrity. The effect of isothermal sintering temperatures (1000 °C to 1200 °C), binder saturation rates, presence of PMMA PSH on the porosity, pore sizes, pore openness index, and mechanical properties of the porous SS316L parts are investigated.

## 2. Materials and Methods

### 2.1. Feedstock

**Figure 1:**
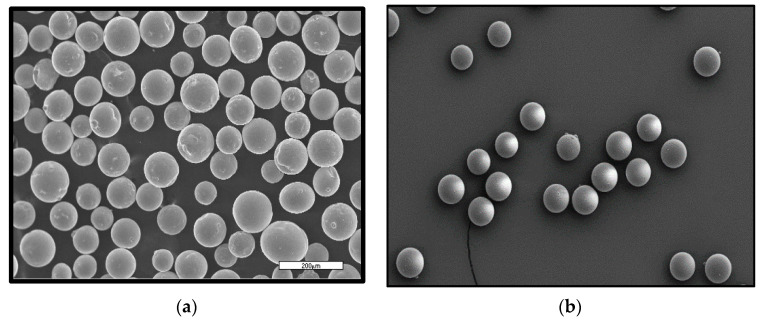
Morphology of (**a**) SS316L and (**b**) PMMA powders.

In the current study, 3D printing of porous 316L stainless steel (SS316L) parts was accomplished by using two types of feedstock: (1) pure SS316L, and (2) SS316L + 30 vol. % PMMA. SS316L + 30 vol. % PMMA feedstock was prepared by dry mixing the required quantities of SS316L and poly (methyl methacrylate) or PMMA powders. Gas atomized SS316L powders of size range 20–53 µm with average particle size of 25.9 µm supplied by Högonäs (Bruksgatan, Sweden), was used as the base material and the powder’s chemical composition are discussed in Table 2. Mono-sized PMMA powder of diameter ~30 ± 0.1 µm supplied by EPRUI Nanoparticles and Microspheres Co. Ltd. (Nanjing Jiangsu, China), was used as the space holder material. SEM analysis of both SS316L and PMMA powders conducted by using a field emission scanning electron microscope (FESEM, Zeiss, Oberkochen, Germany) indicate spherically shaped powders with minimal powder satellites as shown in Figure 1. The particle size of PMMA powders was chosen to be within the powder size range of the base material (SS316L) to minimize the size effects on the powder segregation during recoating process and subsequent layered manufacturing.

### 2.2. Feedstock Characteristics

Feedstock density and flow properties majorly influence the powder-recoating process during additive manufacturing. Hall flow rate can be used to evaluate powder’s flowability and is measured from the time taken by allowing 50 g of powders to pass through a flow funnel consisting of an orifice of size 25.4 mm. Hall flow rate measurements were performed as per ASTM B213-17 [32].

Apparent density measurements were conducted as per ASTMB212-17 [33]. Initially, the feedstock powders were let to freely flow through a flow funnel (without any force) and were filled into a nominal density cup with a standard volume of 25 cm^3^. Later, the excess powders were levelled for the powders to precisely fill the density cup volume. Finally, the apparent density of powders was computed from the mass of the powders within the nominal density cup divided by the volume of cup (25 cm^3^). Tapped density measurements followed ASTMB527-15 [34]. For the tapping experiments, initially the accurately weighed powders were filled within a graduated funnel of known volume. Later, the graduated funnel containing the powder samples was mechanically tapped up to 3000 tap counts at a constant tap frequency of 300 taps/min. Finally, the tapped density of the powders was computed from the mass of the powders within the graduated funnel divided by the final tap volume. True density measurements followed ASTM B923-16 [35] by using an AccuPyc II 1340 helium gas displacement pycnometry system (Micromeritics, Norcross, GA, USA). Powder morphology or powder shape of the feedstock was investigated with a Zeiss field emission scanning electron microscope.

### 2.3. Three-Dimensional Printing of High Porosity 316L Stainless Steel Via Binder Jetting

**Figure 2:**
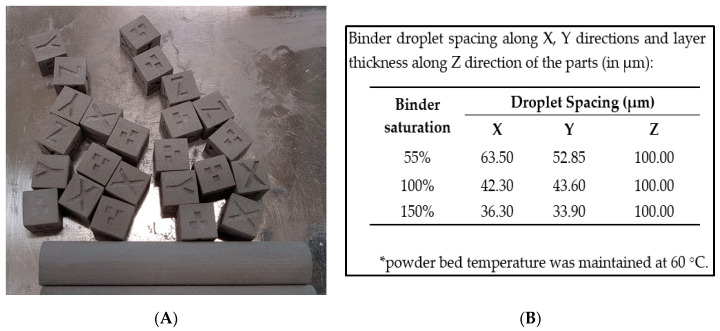
(**A**) As-printed SS316L parts (with and without PMMA) fabricated at 3 levels of binder saturation rates, with X, Y and Z direction markings on their faces that follows 3D printing and sintering directions successfully depowdered and removed from the job box, and (**B**) printing parameters utilized in this study.

An Innovent type binder jetting 3D printer (ExOne, North Huntingdon, PA, USA) with a proprietary aqueous based binder from ExOne (ExOne, North Huntingdon, PA, USA) was utilized for the fabrication of porous SS316L parts. The 3D part details are shown in Figure 2: (1) cubes of dimensions 10 × 10 × 10 mm^3^ with X, Y and Z letter markings on their faces that follows the 3D printing and sintering directions, and 1, 2, and 3 number markings on the surfaces that represents the three different binder saturation rates such as 55%, 100% and 150% employed during 3D printing, respectively, and, (2) cylinders of dimensions 12.5 mm diameter and 80 mm length.

The feedstock powders such as pure 316L stainless steel and 316L + 30 vol. % PMMA were successively added to the powder bed with layer thickness set to 100 µm throughout the experiments. A print head dispensed aqueous-based binder layer wise depending on the input cross-section of the parts received from the STL file by using three different binder saturation rates such as 55%, 100% and 150%, respectively. During binder jetting, the powder bed consists of conditionally packed stainless-steel powders, void spaces or air, and binders. Binder saturation rate is the ratio of volume of binders used during 3D printing to successfully fabricate a solid part to volume of air in the powder bed and is given by Equation (1):

(1) $$\mathrm{Binder}\;\mathrm{saturation}\;\mathrm{rate}=\frac{V_{binder}}{V_{air}\times V_{solid}}==\frac{\mathrm{Volume}\;\mathrm{of}\;\mathrm{binder}}{\left(\left(1-\frac{\mathrm{PR}}{100}\right)\right)\times X\;\mathrm{spacing}\times Y\;\mathrm{spacing}\times Z}$$

where PR is the packing ratio of the powder bed (tapped density of SS316L powders was used in the present study), X and Y spacing are the binder droplet spacing along the XY plane, and Z is the layer thickness (set to 100 µm). The corresponding values of X and Y droplet spacing for the set binder saturation rate are discussed in Figure 2B. The volume of binders was experimentally found by jetting the binders for the set saturation rate on a sponge and measuring its weight. From the experimentally measured binder weight and available binder density values, the volume of binders for a set saturation rate was computed. Later, the computed binder volume was used to verify the set saturation rate by using Equation (1). The as-built parts were cured at 200 °C for 12 h followed by thermal debinding at a peak debinding temperature of 800 °C (for 2 h) and sintering at three different conditions such as 1000 °C, 1100 °C and 1200 °C (for 2 h, each) under high vacuum (≤1 mTorr) and at partial pressure of Ar using a Solar Manufacturing high vacuum furnace (Pennsylvania, USA). A constant heating/cooling rate of 5 °C/min was employed during the sintering cycles. The sintered parts were then used for characterization studies.

### 2.4. Part Characterization

Density/porosity values of the sintered SS316L parts were determined by immersion method following Archimedes principle with de-ionised water as the immersion medium. The total porosity (P), open porosity (Po) and pore openness index (POI) were calculated according to the equivalents Equations (2)–(4), respectively [25]:

(2) $$P=\frac{\rho_{\mathrm{th}}-\rho_{\exp}}{\rho_{\mathrm{th}}}\times 100$$

(3) $$P_{O}=\frac{m_{2}-m_{1}}{m_{2}-m_{3}}\times 100$$

(4) $$\mathrm{POI}=\frac{P}{P_{O}}\times 100$$

where, $\rho_{\mathrm{th}}$ is the theoretical density of SS316L (8 g/cm^3^), $\rho_{\exp}$ is the experimental density of 3D printed SS316L part, $m_{1}$ is the dry weight of part, $m_{2}$ is weight of part that fully infiltrated with de-ionised water, $m_{3}$ is the weight of part in de-ionised water.

The dimensional accuracy of the green parts after 3D printing and the shrinkages in the lateral (diameter) and longitudinal (length) directions after sintering were evaluated by using a Vernier caliper. The lateral (diameter) shrinkage was calculated according to Equation (5):

(5) $$\theta_{\mathrm{lat}}=\frac{D_{0}-D}{D_{0}}\times 100$$

where, $D_{0}$ and D denotes the diameters of SS316L parts before and after sintering. The longitudinal shrinkage values were calculated similarly considering the shrinkage of the length before and after sintering.

The porosity and pore size of the SS316L parts were further investigated by using AutoPore V-Mercury intrusion porosimetry (Micrometrics). Initially, the parts were oven dried at 105 °C (12 h). During the test, mercury invades the pores of the parts with the applied pressure and the corresponding parts’ pore information such as pore sizes and porosity were obtained. Based on the cylindrical capillary model, by assuming the pores to be cylindrical, Washburn equation [36] was used to calculate the pore radius as shown in Equation (6):

(6) $$\Delta P=-\frac{2\;\gamma \;\cos\theta }{R}$$

where, ΔP denotes the pressure (dynes/cm^2^), γ denotes the surface tension of Mercury (485 dynes/cm), θ is the wetting contact angle of mercury (130°) and R is the capillary radius (cm) at the certain pressure.

The fabricated SS316L parts were metallographically polished and were characterized for microstructural investigations with an optical microscope (Olympus, Tokyo, Japan). The ImageJ 1.52n software (NIH, MD, USA) was used to identify the pore fraction (2D porosity information) of SS316L parts (P, in %) using image analysis and the pores in the micrographs were also identified [37]. The chemical composition of the feedstock SS316L powders and as-sintered SS316L parts fabricated by using two types of feedstock (Table 2) were analyzed by Optima 4300 DV (PerkinElmer, Waltham, MA, USA) inductively coupled plasma optical emission spectroscopy, combustion-infrared absorbance (Eltra CS800 Carbon/Sufur Analyzer, Dusseldorf, Germany), inert gas fusion-infrared absorbance and inert gas fusion-thermal conductivity (Eltra ONH 2000 Oxygen/Nitrogen/Hydrogen analyzers) as per CSP-017 Rev. E (ICP-OES), and ASTM E 1019-18. The chemical analysis tests were repeated three times per feedstock type to ensure consistency. The dynamic Young’s modulus of the porous SS316L parts was evaluated at room temperature by using impulse excitation technique with a resonant frequency damping analyzer (ICME, Genk, Belgium) as per the ASTM E1876-15 [38]. Parts of 12 mm diameter and 80 mm length (l/d > 6) were used for the characterization. Compression properties of the parts were tested by using a 5982 Universal Testing System (Instron Norwood, MA, USA) at a strain rate of 7 × 10^−4^ s^−1^ (crosshead speed of 0.5 mm/min) according to ASTM E9-19 [39]. Parts with 12 mm diameter and 12 mm length (l/d = 1) were used for the compression test. Porosity measurements and compression experiments were repeated at least 5 times to ensure result consistency.

## 3. Results and Discussion

### 3.1. Feedstock Characteristics

The density and flowability characteristics of the feedstock are discussed in Table 3. The results indicated that with the addition of 30 vol. % PMMA, the Hall flow rate and apparent density values of the SS316L powders were found to be affected and this is attributed due to the inherent cohesive nature of PMMA polymeric fine powders of size 30 µm. SS316L + 30 vol. % PMMA exhibited poor Hall flow rate of 28 s 11′ (50 g^−1^), apparent density of 3.054 g/cm^3^, and apparent packing factor (p.f) of 51.3% when compared to that of the pure SS316L feedstock with Hall flow rate of 18 s 18′ (50 g^−1^), apparent density of 4.601 g/cm^3^, and apparent p.f of 58.02%, respectively. The apparent density of powders drop along with the growth of interparticle friction forces and this is due to the prevailing high resistance of SS316L particles containing PMMA to re-arrange during their apparent flow leading to poor powder packing and flowabilty characteristics [40]. Upon tapping, the density of SS316L + 30 vol. % PMMA was found to improve exhibiting tapped density of ~3.720 g/cm^3^ and tapped p.f of 62.48% which is slightly greater than that of the pure SS316L feedstock (62.15%) indicating the possible re-arrangement of low density (1.18 g/cm^3^) and fine PMMA powder particles (30 µm) filling the interstitial powder spaces. Hausner ratio (HR) is the ratio of tapped density to apparent density of powders [40]. The significant decrease in the apparent density of SS316L + 30 vol. % PMMA feedstock led to increase in the HR value to ~1.2. Powders with HR ratio > 1.5 are classified as non-freely flowing with fluidization problems [41]. Both the pure SS316L and SS316L + 30 vol. % PMMA feedstock are classified as freely flowing based on their HR values (HR < 1.5, Table 3) and pure SS316L feedstock exhibited HR value as low as ~1 indicating excellent flowability.

### 3.2. Part Characteristics

#### 3.2.1. Effect of Binder Saturation Rates on The Dimensional Accuracy of The as-Printed SS316L Parts

**Figure 3:**
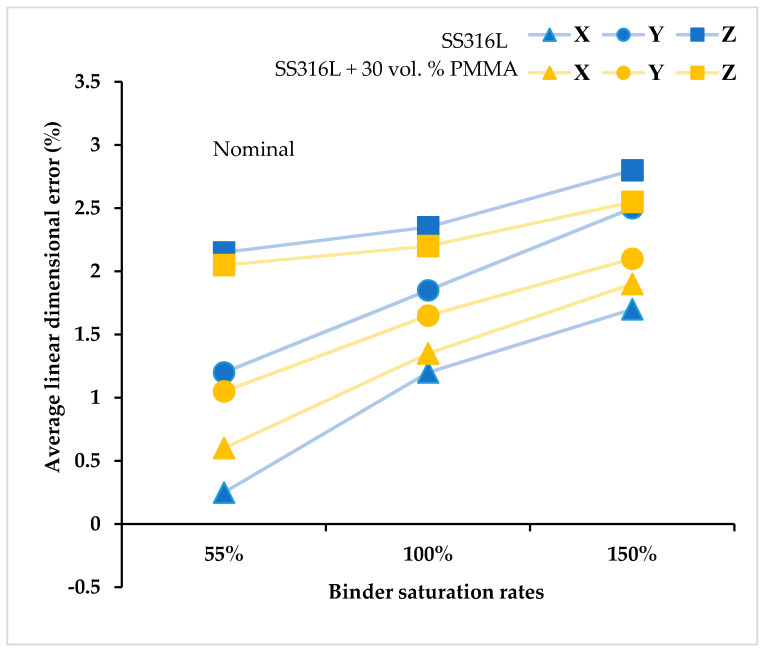
Dimensional accuracy results of SS316L green samples.

Figure 3 shows the dimensional accuracy results of the as-printed green SS316L parts (10 × 10 × 10 mm^3^) measured right after the 3D printing process fabricated by using different binder saturation rates and at a constant layer thickness value set to 100 µm. The dimensions of the as-printed green parts were found to be higher than the 3D model dimensions used during printing irrespective of the binder saturation rates. Further, the printing directions influence the dimensional accuracy of the green parts. The dimensions of the parts along the X and Y printing directions are majorly controlled by the binder droplet spacing and their corresponding values for the set binder saturation rates are discussed in Figure 2. Low binder saturation rates lead to insufficient binders to firmly join or bond the metal powders together causing pre-mature failure of the as-printed green parts during depowdering and subsequent handling for post-processing steps. In the present study, all the SS316L green parts maintained good structural integrity and did not fail during handling and subsequent sintering steps indicating sufficiently bound SS316L powder particles even at a low binder saturation rate of 55%.

$$\mathrm{Linear}\;\mathrm{dimensional}\;\mathrm{error}\;\left(\%\right)=\frac{3D\;\mathrm{printed}\;\mathrm{part}\;\mathrm{measured}\;\mathrm{dimensions}-\mathrm{input}\;3D\;\mathrm{model}\;\mathrm{dimension}}{\mathrm{input}\;3D\;\mathrm{model}\;\mathrm{dimension}}\times 100.$$

*X, Y and Z directions denote the 3D printing directions. The standard deviation of average linear dimensional error along the X and Y directions was found to be ±0.15% (equivalent to ~ ±0.03–0.05 mm), and along the Z direction, it is 0.35% (equivalent to ~ ±0.07–0.09 mm) for both the feedstock types.

The linear dimensional error along the Z direction was found to be the maximum irrespective of the feedstock type and binder saturation rates. This is predominantly due to the combined effects of: (1) selection of layer thickness value of 100 µm, and (2) different capillary mediated binder infiltration rates along the X, Y, and Z directions of the part due to the heterogeneous porosity within the packed powders arising during powder layering and subsequent printing due to differences in the binder drop spacing, layer thickness and powder size. Further, an increase in the binder saturation rates increases the dimensional error or decreases the dimensional accuracy of the parts along the printing directions. Similar observations w.r.t poor dimensional accuracy with increase in the binder saturation rates and along the Z printing direction of the binder jet parts was previously reported by Xia et al. [42]. Poor dimensional accuracy at higher binder saturation rates is due to the bleeding or unintended spread of binders outside the print area that bond excess or unnecessary powders to the part surfaces or migrate the part surface slightly outwards affecting its dimensional accuracy [42]. Low binder droplet spacing will cause over-saturation and excessive adhesion between the powders [43]. There exists an optimum binder droplet spacing under which the printed lines will be smooth, narrow and more uniform, and the representative 3D printed green parts exhibit smallest dimensional error [44]. In the present study, the green parts printed at 55% binder saturation rate exhibited relatively better dimensional accuracy for both the feedstock types.

#### 3.2.2. Results of Porosity Measurements

**Figure 4:**
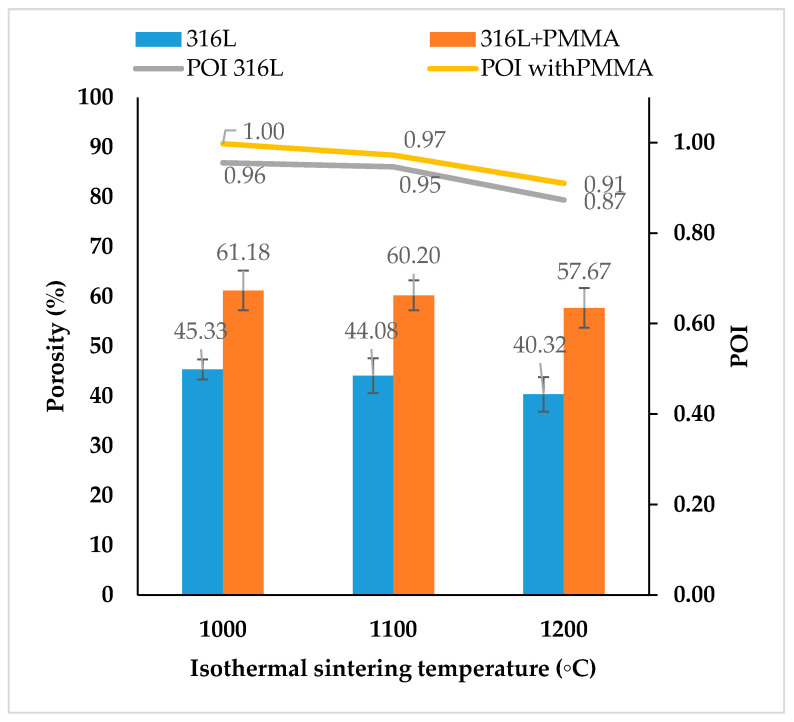
Porosity results of SS316L parts sintered at 1000 °C, 1100 °C and 1200 °C using pure SS316L, and SS316L + 30 vol. % PMMA feedstock fabricated at 55% binder saturation rate. Note: Porosity of SS316L parts was measured by using the Archimedes (water immersion) method.

The porosity values of binder jet SS316L parts were measured by using the Archimedes principle (water immersion method) and further confirmed by mercury intrusion porosimetry and image analysis of optical micrographs, respectively. A theoretical pure SS316L stainless steel density of 8 g/cm^3^ was used for the porosity calculations. Several factors such as: (1) sintering parameters (isothermal sintering temperature, holding time, and heating rate), (2) binder volume controlled by the set binder saturation rates, (3) volume of PMMA space holder particles in the feedstock, and (4) feedstock characteristics, together affect the porosity values of SS316L parts. In the present study, the sintering conditions such as holding time of 2 h, heating and cooling rates of 5 °C/min and sintering atmosphere of high vacuum with partial pressure of argon were kept constant throughout the experiments. Isothermal sintering temperature effects on the porosity values of SS316L parts fabricated at a constant binder saturation rate (set to 55%) using pure SS316L and SS316L + 30 vol. % PMMA feedstock are shown in Figure 4.

With increasing sintering temperatures, the interstitial void spaces between the SS316L powder particles decrease and thereby decrease the pore sizes and porosity of the parts but affect their pore openness index values with presence of possible pore closure within the parts.

For the sintering temperatures between 1000 °C and 1200 °C, porosity values of 40–45% were observed for the parts fabricated by using pure SS316L feedstock and ~57–61% for the parts fabricated by using SS316L + 30 vol. % PMMA feedstock, respectively. The SS316L parts sintered up to 1100 °C exhibited POI of ~1 indicating all the pores to be open and well interconnected. At the sintering temperature of 1200 °C, SS316L parts exhibited POI of ~0.87–0.91 indicating most of the pores to be open. The reduced POI value at 1200 °C is due to the enhanced SS316L powder consolidation during sintering at high isothermal sintering temperature forming strong neck connections and subsequent densification.

**Figure 5:**
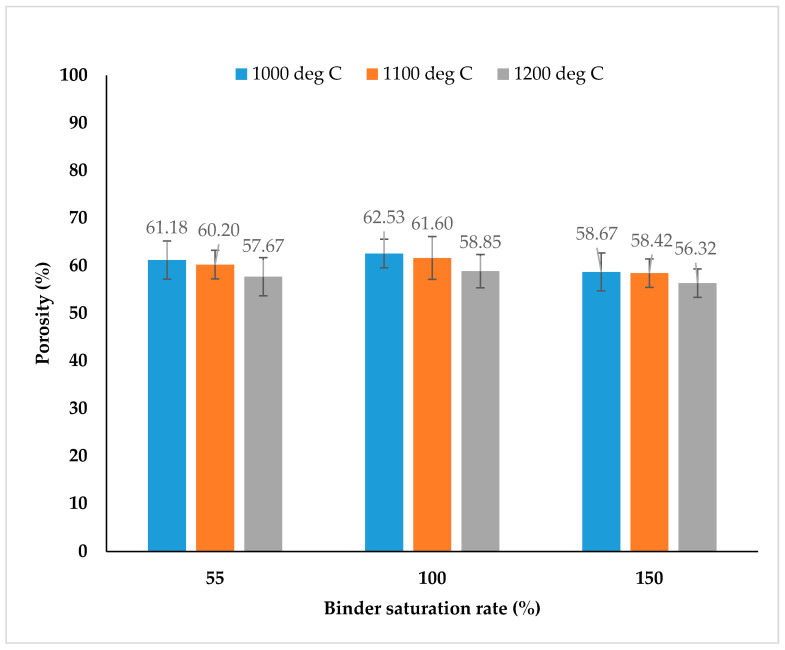
The combined influence of binder saturation rates, sintering temperatures and PMMA space holders on the porosity of SS316L parts fabricated using SS316L + 30 vol. % PMMA feedstock. * Note: Porosity of SS316L parts was measured by using the Archimedes (water immersion) method.

Figure 5 shows the combined influence of different binder saturations rates, isothermal sintering temperatures and presence of PMMA space holders in the 3D printing feedstock on the porosity values of SS316L parts. The binder volume did not contribute much to the porosity values of SS316L parts and with increase in the binder saturation rates (up to 150%), there was only a feeble change (by ±2%) in the porosity of the parts.

Porosity changes (by ±2%) are attributed to possible changes in the powder packing during 3D printing as a result of rearrangement of powder particles on the powder bed during powder recoating and subsequent binder jetting with changes in the X and Y binder droplet spacing and thereby causing changes in the binder penetration behavior into the packed powder bed (Figure 2). Lighter and mono-sized PMMA particles with density of ~1.18 g/cm^3^ and size of 30 µm are highly prone to become rearranged due to powder segregation effects during the powder recoating process and infiltration of binders into packed powders every layer [45]. The density of PMMA is closer to the density of aqueous binder (~0.9–1 g/cm^3^), but there is a strong mismatch in the density values between PMMA and SS316L (~8 g/cm^3^).

**Figure 6:**
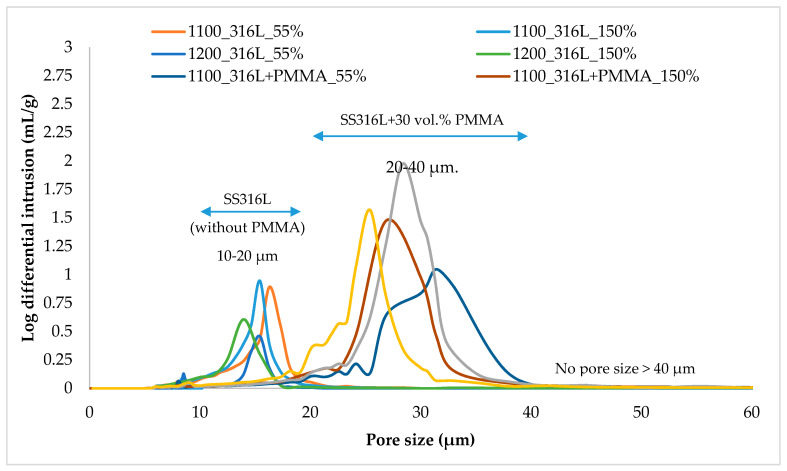
Representative Mercury intrusion porosimetry results of SS316L parts indicating the average pore sizes. * Note: part name format follows sintering temperature_feedstock_binder saturation rate. For example: 1100_316L_55% denotes SS316L parts sintered at 1100 °C fabricated using 55% binder saturation rate using pure SS316L feedstock.

The results of porosity and average pore size of SS316L parts measured by using the mercury intrusion method are discussed in Table 4 and Figure 6. For comparison purpose, the parts fabricated by using the lowest (55%) and the highest (150%) binder saturation rates were studied. Pore sizes of parts fabricated by using pure SS316L feedstock were found to be in the range of 10–20 µm, whereas parts fabricated by using SS316L + 30 vol. % PMMA feedstock exhibited a bigger pore size range of 20–40 µm, respectively. This increase in the pore size is attributed due to the decomposition of 30 µm PMMA powder particles used as space holder material leaving behind bigger pores of size ≥30 µm. No pore size greater than 40 µm was observed.

**Figure 7:**
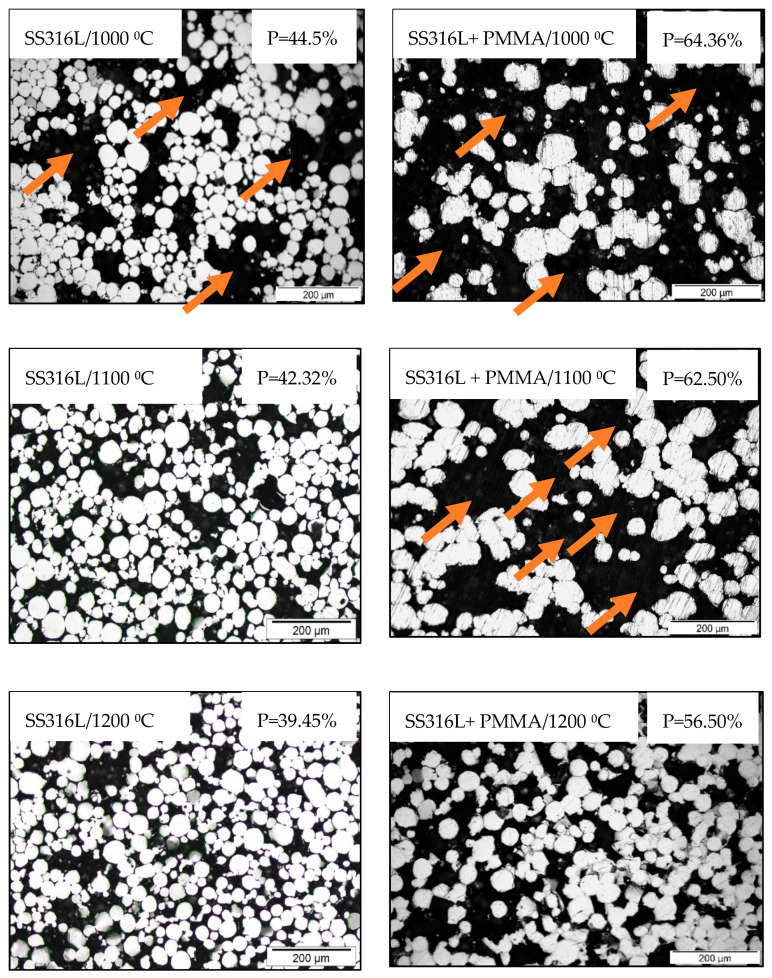
Optical micrographs of porous SS316L parts revealing 2D porosity information. * Note: Binder saturation rate for all the parts was set to 55%. The images were sampled from the cylindrical coupons (Figure 2) at locations closer to its center. The arrow marks indicate presence of big voids within the parts leading to insufficient particle necking.

Optical micrographs of SS316L parts revealing the relative 2D porosity information and the results of pore fraction measured by image analysis are shown in Figure 7. Further, the microstructure images revealed the presence of big voids within the porous SS316L parts fabricated by using pure SS316L feedstock sintered at 1000 °C and for the other SS316L parts fabricated by using SS316L + 30 vol. % PMMA feedstock sintered at 1000 °C and 1100 °C, respectively.

The porosity values measured by using the mercury intrusion method (Table 4) and pore fraction values by image analysis of optical micrographs (Figure 7) were found to be in consensus with those measured by using the Archimedes method. The binder saturation rate was found to have no significant influence on the porosity of parts fabricated using pure SS316L feedstock. The presence of PMMA in the feedstock led to decrease in the porosity with increasing binder saturation rates and this behavior was found to be in consensus with the previous study on binder jetting of PMMA which is due to the interaction between the binder phase and PMMA [46].

Table 5 discusses the porosity values of SS316L and austenitic steel parts fabricated by conventional and selective laser sintering processes. The results indicate that finer pores with controlled pore size and pore interconnectivity are able to be achieved by binder jetting with feedstock containing space holders proposed in the present study.

#### 3.2.3. Results of Chemical Analysis

Keeping the carbon (C), hydrogen (H) and oxygen (O) contents to the lowest levels throughout the binder jetting and subsequent sintering processes is of paramount importance especially for the successful processing of low carbon austenitic stainless steel SS316L to ensure its superior corrosion and mechanical properties. Table 2 presents the chemical composition results of the as-received SS316L staring powders and final sintered SS316L parts fabricated using different binder saturation rates with two types of feedstock.

The results indicated that the chemical composition of the final sintered SS316L parts do not change throughout the processing and exactly matches to that of the starting SS316L powders for 55% binder saturation rate. At 150% binder saturation, there is no change in the chemical composition of the final parts fabricated with pure 316L feedstock. Both the binder phase and PMMA materials consist of C and H as the major constituents and due to which the parts fabricated using SS316L + 30 vol. % PMMA (at 150% binder saturation) suffer from increase in the C content to 0.07 wt. %, but matches the composition of SS316. No change in the C, H and O composition confirms the binder jetting processing route (present study) to be contamination-free and ideal for fabricating porous 316L stainless steel and an optimum binder saturation rate (for example, 55% in the present study) and right selection of binder phase and space holder materials will further help to avoid contamination especially for C sensitive materials like SS316L stainless steel.

#### 3.2.4. Results of Shrinkage Measurements

**Figure 8:**
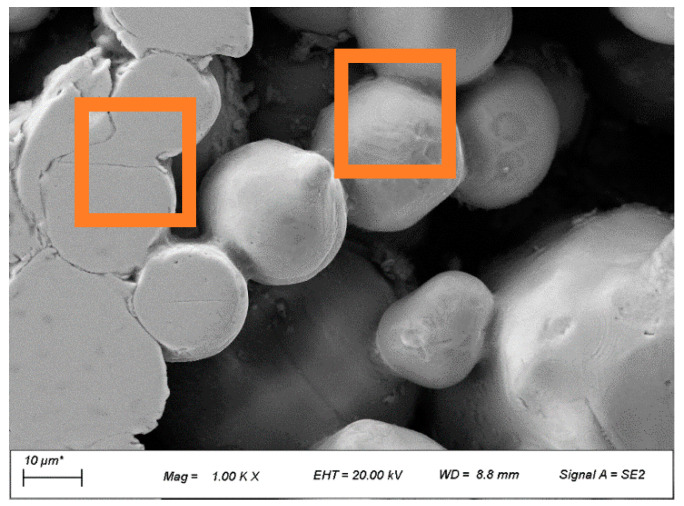
Representative evidence of interparticle necking observed in SS316L + 30 vol. % PMMA parts sintered at 1200 °C. * Note: Marked area shows neck regions.

For the SS316L porous parts to exhibit good mechanical properties, strong interparticle necking between the powder particles should initiate during sintering for which SS316L atoms can transport from the interior of the part (bulk transport phenomenon or volumetric diffusion) and from the surface (or surface phenomenon) to fill the vacant pore sites around the particle contacting points to form necks that subsequently shrinks the part. Figure 8 shows representative evidence of interparticle necking for high porosity SS316L parts fabricated using SS316L + 30 vol. % PMMA feedstock and sintered at 1200 °C.

**Figure 9:**
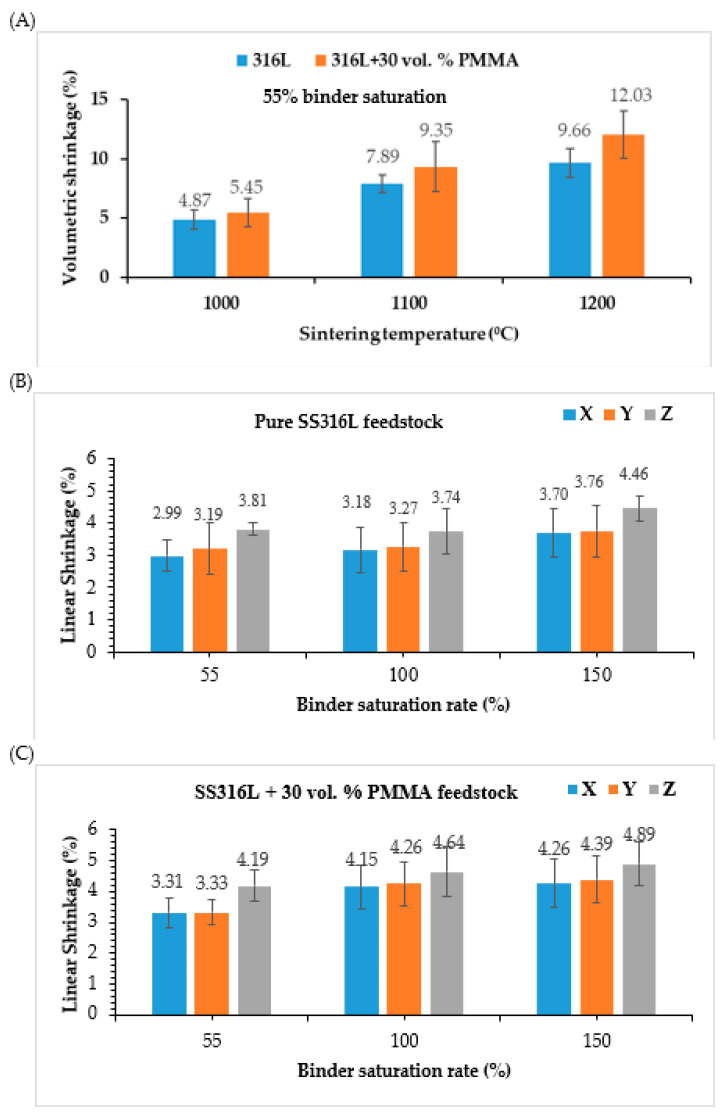
Results of (**A**) volumetric shrinkage measurements (at 55% binder saturation rate), and linear shrinkage measurements at 1200 °C of SS316L parts fabricated using (**B**) Pure SS316L, and (**C**) SS316L + 30 vol. % PMMA feedstock.

The effects of isothermal sintering temperatures on the volumetric shrinkage values of SS316L parts are shown in Figure 9A. As expected, the shrinkage grows with increasing isothermal sintering temperature and maximum volumetric shrinkage values of ~9.66% and ~12% were observed for the SS316L parts sintered at 1200 °C fabricated by using pure SS316L and SS316L + 30 vol. % PMMA feedstock types, respectively. The shrinkage in the parts are predominantly due to the SS316L powder consolidation during sintering at higher isothermal sintering temperatures and partially due to the decomposition of binders and PMMA [6]. After the decomposition of 30 vol. % PMMA from the SS316L parts, there were only less SS316L powders surrounding the spaces (or presence of bigger voids) that inhibit the network formation between the powders. Apart from the isothermal sintering temperature, the shrinkage of the SS316L sintered parts also depends on their initial powder packing before sintering that affects the part porosity as the reduction of micro-pore sizes within the parts during sintering majorly contribute to the shrinkage of the high porosity parts [47]. Feedstock density characteristics affect the powder packing. By using pure SS316L feedstock exhibiting higher density characteristics (Table 3), the powder packing within the parts can be substantially improved and thereby can mitigate shrinkage and similar behavior was observed for SS316L parts fabricated by metal injection moulding with feedstock containing SS316L nanoparticles without space holders contributing to their particle packing density and thereby exhibiting low shrinkage values [47].

For the same sintering conditions, SS316L parts (sintered at 1200 °C) exhibited higher shrinkage values at high binder saturation rates (150%) when compared to the parts fabricated at low binder saturation rates (55%) for all the X, Y and Z directions (Figure 9). Further, the shrinkage values of SS316L parts fabricated with SS316L + 30 vol. % PMMA feedstock was found to be significantly high when compared to that of the parts fabricated with pure SS316L feedstock and the results are consistent to the previous works by Ziaee et al. [48] confirming that parts consisting of less pore formers possesses lower shrinkage.

The mismatch or differences in the linear shrinkage values along the X, Y and Z directions of the part or presence of anisotropic shrinkage is predominantly due to: (1) non-uniform binder droplet spacing along the X, Y directions arising during 3D printing that are majorly controlled by the binder saturation values (Figure 2), and (2) set layer thickness value (100 µm) that alters the spacing in the Z direction affecting the powder packing within the green part. In the present study, shrinkage anisotropy along the Z direction was found to be the maximum. The shrinkage values along the X and Y directions of the part were found to be relatively more uniform especially at 100% binder saturation rate and this is attributed due to almost equal-sized X and Y binder droplet spacing (~43 µm). Further, the linear shrinkage values were found to be less than 5% indicating surface diffusion phenomenon as the predominant mechanism. This is also supported by the Figure 8 and the theory suggesting that the particle necking should be greater than 1/3^rd^ of the particle diameter to realize volumetric diffusion [49,50].

#### 3.2.5. Results of Dynamic Young’s Modulus and Compression Properties

**Figure 10:**
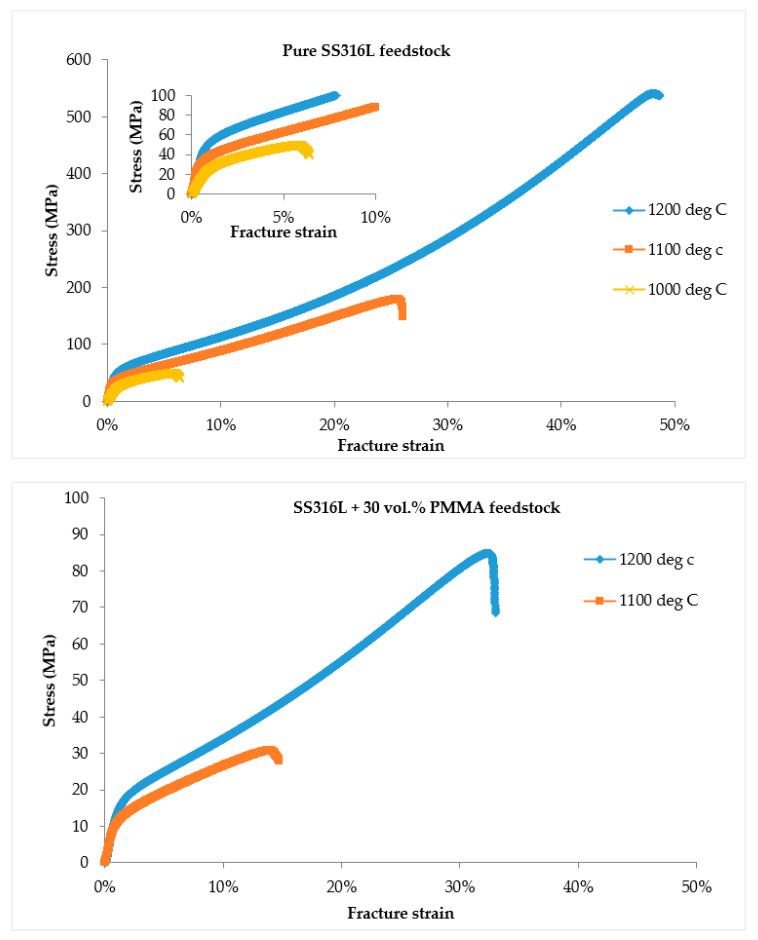
Stress-strain curves during compression loading of porous SS316L parts.

Table 5 presents the dynamic Young’s modulus and compression property results of the binder jetting-processed porous SS316L parts and Figure 10 shows the representative stress-strain curves under compression loading, respectively. The dynamic Young’s modulus of the SS316L stainless steel parts decreased with increasing porosity values and different Young’s modulus values in the range of 2–29 GPa were able to be achieved with changes in the porosity of the parts. The stress-strain curves observed during compression loading of porous parts can be generally categorized into three distinct regions [51]: (1) within the elastic regime, stress increases linearly with strain, (2) followed by a long deformation plateau with a small increase of flow stress to large strain, and (3) a final densification stage where the flow stresses rapidly increase resulting to fracture. At low stress values, all the stress-strain curves of binder jetting processed porous SS316L parts exhibited a very similar behavior under compression where the stresses raised almost linearly with strain (or elastic deformation). The SS316L parts fabricated by using pure SS316L feedstock sintered at 1200 °C exhibited the maximum 0.2% compressive yield strength (0.2% CYS) of 52.7 MPa which is almost 50% (34.7 MPa) and 100% (26.2 MPa) greater than the other binder jet SS316L parts fabricated by using pure SS316L feedstock sintered at 1100 °C and 1000 °C, respectively. The 0.2% CYS was found to be significantly affected for the parts containing high porosity values (~60%) fabricated by using SS316L + 30 vol. % PMMA feedstock exhibiting 0.2% CYS values of only 12.6 MPa and 16.2 MPa when sintered at 1100 °C and 1200 °C, respectively. Specific compressive strength is the ratio of 0.2% CYS to the density of material. Specific compressive strength decreased with the increasing porosity of the parts. High specific compressive strength of ~11 MPa/(g/cm^3^) was observed for the parts fabricated with pure SS316L feedstock sintered at 1200 °C.

Beyond the elastic regime, the deformation plateau significantly varied with porosity. The parts fabricated by using pure SS316L feedstock sintered at 1100 °C and 1200 °C with porosity of 44% and 40%, respectively, exhibited a long deformation plateau followed by densification where the flow stresses increased rapidly achieving a significant ultimate compressive strength (UCS) values of 172 MPa (for 1100 °C) and 520 MPa (for 1200 °C), respectively, and the corresponding fracture strain (FS) values were ~24% (for 1100 °C) and ~36.4% (for 1200 °C), respectively. Work of fracture or energy absorption of a material is found from the surface below the stress-strain curve and it was found to be the maximum of ~116.7 MJ/m^3^ for SS316L parts fabricated with pure SS316L feedstock sintered at 1200 °C indicating higher capability to absorb energy until fracture upon compressive loading. But, the SS316L parts sintered at 1000 °C exhibiting porosity of 45.3% fabricated by using pure SS316L feedstock failed with UCS and FS values of ~47 MPa and ~5.1%, respectively, indicating poor consolidation of SS316L powders during sintering leading to insufficient or weak particle necking that was revealed during microstructural characterization with micrographs indicating presence of big voids (Figure 7). Similar weak behavior was observed for the high porosity (~60%) SS316L parts fabricated by using SS316L + 30 vol. % PMMA feedstock and the parts exhibited UCS of only 35 MPa and 75.4 MPa when sintered at 1100 °C and 1200 °C, respectively, and the corresponding FS values were 13.2% (for 1100 °C) and 27.3% (for 1200 °C). The SS316L parts fabricated by using SS316L + 30 vol. % PMMA sintered at 1000 °C were very fragile and failed at very low compressive stresses upon loading.

The compression properties of high porosity metals follow the Gibson and Ashby model [52]. The relative density of porous metals is the most significant structural property that influences the stresses upon loading and is given by $\frac{\rho_{exp}}{\rho_{Solid}}$; where, $\rho_{exp}$ is the experimental density of porous SS316L parts and $\rho_{Solid}$ is the theoretical density of solid (fully dense SS316L stainless steel with density of 8 g/cm^3^). The relationship between relative stress, Young’s modulus and relative density are calculated according to Equations (7) and (8), respectively [52]:

(7) $$\frac{0.2{\;\%\;\mathrm{CYS}}_{\mathrm{Exp}}}{0.2{\;\%\;\mathrm{CYS}}_{\mathrm{Solid}}}=C1\times {\left(\frac{\rho_{\exp}}{\rho_{\mathrm{Solid}}}\right)}^{\frac{3}{2}}$$

(8) $$\frac{E_{\mathrm{Exp}}}{E_{\mathrm{Solid}}}=C2\times {\left(\frac{\rho_{\exp}}{\rho_{\mathrm{Solid}}}\right)}^{2}$$

where, $0.2{\;\%\;\mathrm{CYS}}_{\mathrm{Exp}}$ is the experimental 0.2% CYS of porous SS316L parts (present study), $0.2{\;\%\;\mathrm{CYS}}_{\mathrm{Solid}}$ is the nominal 0.2%CYS of fully dense SS316L solid (172 MPa) [53], $E_{\mathrm{Exp}}$ is the experimental Young’s modulus of porous SS316L parts (present study), $E_{\mathrm{Solid}}$ is the nominal Young’s modulus of fully dense stainless steel (193 GPa) [53]. C1 and C2 are positive constants that mainly depend on the pore structures [19].

**Figure 11:**
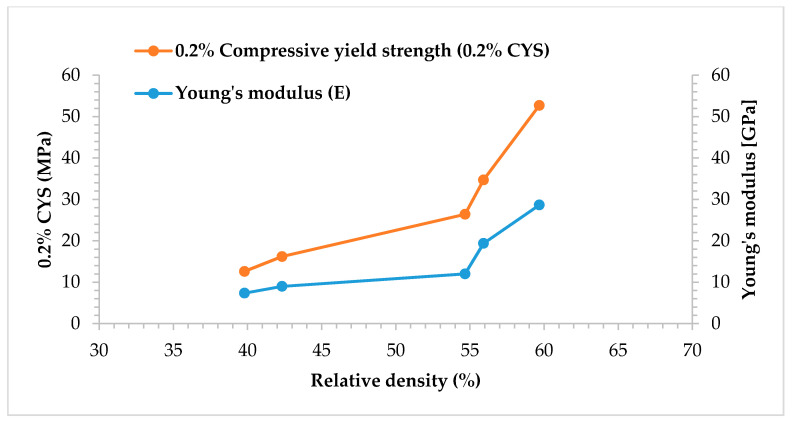
Relative density versus Young’s modulus (E) and 0.2% compressive yield strength (0.2% CYS).

The relationships between $0.2{\%\mathrm{CYS}}_{\mathrm{Exp}}$, $E_{\mathrm{Exp}}$, and the relative densities of binder jetting processed SS316L parts are shown in Figure 11. Both the 0.2% CYS and E of the porous parts increased with increasing relative density (or decreasing porosity) as observed with the other studies on porous metals [54]. The significant increase in the overall compression properties observed with the parts fabricated by using pure SS316L feedstock sintered at 1100 °C and 1200 °C led to a sudden upward shift in the E and 0.2% CYS versus relative density curves (Figure 11). This upward shift or sudden increase in the slope of the curve indicates significant powder consolidation and formation of strong interparticle necking between SS316L powders during sintering exhibiting sudden increase in the 0.2% CYS and Young’s modulus values when compared to the other SS316L parts. The findings are in consensus with the compressive stress-strain curves (with long deformation plateau) and microstructural investigations (Figure 7) indicating absence of voids for SS316L parts sintered at 1100 °C and 1200 °C unlike other SS316L samples.

The change in slope of the curves (Figure 11) indicate that the constants C1 and C2 from Equations (7) and (8) significantly rely on the interparticle necking between 316L powders during sintering but the dependency of the constants on the SS316L porous structures are not well understood especially for high porosity parts as reported in the previous study [19].

The compression properties of the fabricated SS316L porous parts are compared to that of the cancellous bone types such as Femoral head, Femoral condyle and vertebra [55]. The results (Table 5) indicate that the compression properties of SS316L parts are closer to that of the cancellous bone types and especially matches with the Young’s modulus values. Table 5 lists the compression properties of several porous SS316L parts fabricated by conventional processes. The properties achieved in the present study are still comparable to the other conventionally processed porous SS316L parts and even surpasses those having similar porosities.

The present work offers more insights into correlation between porosity and respective Young’s modulus and compression properties of the binder jetting processed SS316L stainless steel parts and provides range of properties to target different applications as per the requirements, with parts having open pores and a controlled pore size of <40 µm. In future work, corrosion studies and effect of varying layer thicknesses and different X and Y binder droplet spacing processing parameters on the porosity and shrinkage anisotropy of binder jet processed SS316L parts will be investigated.

## 4. Conclusions

High porosity 316L stainless steel (SS316L) with total porosity of ~40–60% and pore openness index of 0.87 to 1 were successfully fabricated by binder jetting and subsequent sintering (up to 1200 °C) coupled with the powder space holder (PSH) technique by using 30 µm equal-sized PMMA powders as PSH. Two approaches have been systematically studied to understand their effects on the porosity of binder jet parts; (1) pores-by-processing approach by varying the isothermal sintering temperatures (1000 °C, 1100 °C and 1200 °C for 2 h, each) and binder volumes at different binder saturation rates (55%, 100% and 150%), and (2) pores by feedstock modification approach by adding PSH (30 vol. % PMMA) to pure SS316L feedstock.

The following are the primary conclusions of the present study:

- Isothermal sintering temperature plays a vital role in controlling the porosity of SS316L parts; porosity increased with decreasing sintering temperatures, whereas varying binder saturation rates affected the porosity values by only ±2%. Through pores-by-processing approach (present study), porosity of 40%–45% was achieved.
- With the addition of 30 vol. % of PMMA powders to the SS316L feedstock, the porosity values of parts sintered up to 1200 °C (2 h, each) increased significantly to 57%–61%.
- Mercury porosimetry results indicated pore sizes of 10–20 µm for the parts fabricated with pure SS316L feedstock and 20–40 µm for the parts fabricated with SS316L + 30 vol. % PMMA feedstock, respectively, with no pore size >40 µm.
- All the parts exhibited anisotropic shrinkage especially along the Z direction predominantly due to the mismatch between the set layer thickness (100 µm) and X & Y binder droplet spacing that varies based on the set binder saturation rates.
- The dynamic Young’s modulus and compression properties of the SS316L stainless steel parts increased with increasing relative density (or decreasing porosity). The SS316L parts fabricated by using pure SS316L feedstock sintered at 1200 °C exhibited the maximum overall compressive properties with 0.2% compressive yield strength of 52.7 MPa, ultimate compressive strength of 520 MPa, fracture strain of 36.4%, and energy absorption of 116.7 MJ/m^3^, respectively.
- Low Young’s modulus values in the range of 2–29 GPa could be achieved. The Young’s modulus and compression properties of the binder jet SS316L parts were found to be on par with that of the conventionally processed SS316L parts and even surpassed those having similar porosities and matched to that of the cancellous bone types.
- The final chemical composition of the sintered SS316L parts exactly matched the chemical composition of starting SS316L powders with no C, H and O contaminations confirming binder jetting process route to be contamination-free and ideal for fabricating porous austenitic 316L stainless. An optimum binder saturation rate of 55% was found to be more favourable to fabricate contamination-free SS316L parts with high dimensional accuracy.

## Figures and Tables

**Table 1:** Literature search results of binder jetting manufactured porous metal parts.

Materials	Space Holder/Foaming Agent	Applications	Printing Systems	Porosity	Pore Interconnectivity	Average Pore Diameter
Fe-30Mn [23]	–	Biomedical	ExOne Lab	36.3%	–	500 µm & 1 mm
Pure Ti [24]	PVA	Biomedical	ZCorp 310 plus	31–43%	–	–
Ti + Mg [25]	PVA	Biomedical	ZCorp 310 plus	3D printed Ti: 41.33%	82.5%	97 ± 26 µm
				Ti + Mg: 7.56%	–	–
Ti-6Al-4V [26]	–	Biomedical	Spectrum Z510	As printed: 52%	99.8%	8.9 µm
				As sintered: 28%	98.4%	12.5 µm
Pure Ti [27]	PVA	Biomedical	ZCorp 310 plus	Ti + 5% PVA: 32.2–52.7%	–	Median 20 µm
Copper [28]	CuO	Electrical, biomedical	ExOne R2	41.6–58.1%	–	–
Pure Ti [29]	Paraffin/wax progen	Biomedical	PIM integrated binder jet 3D printer	35–40%	–	0.37 mm–0.47 mm
Iron [30]	PVA	Biomedical and tooling	ZPrint 310 plus	64.5–91.3%	–	–
CoCrFeNiMn [31]	–	Filtration	Innovent, ExOne	34–40%	87%–89%	1 µm–100 µm

**Table 2:** Chemical composition of as received 316L stainless steel powders and sintered SS316L parts 3D printed by using two types of feedstock processed at two binder saturation rates.

Elements	As Received SS316L Powders	Sintered SS316L Parts Using Two Types of Feedstock			
		55% Binder Saturation		150% Binder Saturation	
		Pure SS316L	SS316L + 30 vol. % PMMA	Pure SS316L	SS316L + 30 vol. % PMMA
C ^1^	0.03	0.03	0.03	0.03	0.07
Mn	1.10	1.09	1.10	1.10	1.09
Si	0.74	0.73	0.74	0.74	0.73
P	0.034	0.036	0.036	0.034	0.036
S ^1^	0.003	0.002	0.002	0.003	0.002
Cr	16.74	16.73	16.73	16.74	16.73
Mo	2.44	2.38	2.38	2.44	2.38
Ni	12.97	12.77	12.77	12.97	12.77
Cu	0.10	0.11	0.11	0.10	0.11
Co	0.04	0.04	0.04	0.04	0.04
Nb	0.01	0.01	0.01	0.01	0.01
V	0.01	0.01	0.01	0.01	0.01
W	0.05	0.05	0.05	0.05	0.05
O ^2^	0.034	0.33	0.033	0.34	0.033
N ^3^	0.001	0.001	0.001	0.001	0.001
H ^3^	0.0013	0.0017	0.0017	0.0013	0.0017
Fe	Balance	Balance	Balance	Balance	Balance

* Note: All results are in weight percent. Other elements tested (<0.01% each) are Al, As, B, Bi, Ca, Cd, Mg, P, Sn, Ta, Ti, Zn, Zr. ^1^ Determined by combustion-infra-red absorbance. ^2^ Determined by inert gas fusion-infrared absorbance. ^3^ Determined by inert gas fusion-thermal conductivity.

**Table 3:** Density and flowability characteristics of as-received SS316L and SS316L + 30 vol. % PMMA feedstock.

Powder Samples	Flowability Characteristics	Density Characteristics (g/cm^3^)			Hausner Ratio, HR (B/A)	Powder Packing Factor p.f (%)	
	Hall Flow Rate (s. 50 g^−1^)	Apparent (A)	Tapped (B)	True (C)		Apparent p.f (A/C) × 100	Tapped p.f (B/C) × 100
As received SS316L powders	18 s 18′	4.601	4.928	7.929	1.071	58.02	62.15
SS316L + 30 vol. % PMMA	28 s 11′	3.054	3.720	5.953	1.218	51.30	62.48

**Table 4:** Porosity and average pore size of SS316L parts measured by the mercury intrusion method.

Part Name	Porosity (%)	Average Pore Size (µm)
1100_316L_55%	42.50 ± 3.0	16.30 ± 1.50
1100_316L_150%	42.70 ± 2.5	15.45 ± 0.70
1200_316L_55%	38.70 ± 2.0	15.45 ± 1.00
1200_316L_150%	38.20 ± 2.0	13.95 ± 1.50
1100_316L+PMMA_55%	60.45 ± 3.0	28.35 ± 3.50
1100_316L+PMMA_150%	58.60 ± 2.5	26.85 ± 2.00
1200_316L+PMMA_55%	58.65 ± 3.0	30.60 ± 3.50
1200_316L+PMMA_150%	56.10 ± 3.5	25.44 ± 2.50

* Note: Part name format follows sintering temperature_feedstock_binder saturation rate. For example: 1100_316L_55% denotes SS316L parts sintered at 1100 °C fabricated using 55% binder saturation rate using pure SS316L feedstock.

**Table 5:** Dynamic Young’s modulus and compression properties of porous SS316L parts.

Part Description	Sintering Temperature [°C]	Total Porosity [%]	Density [g/cm^3^]	Average Pore Size [µm]	Dynamic Young’s Modulus [GPa]	0.2%CYS [MPa]	Specific Compressive Strength [MPa/(g/cm^3^)]	UCS [MPa]	Fracture Strain [%]	Work of Fracture or Energy Absorption [MJ/m^3^]
SS316L (Present study)	1000	45.33	4.3736	16 ± 2	10.52 ± 0.02	26.4 ± 2	6.03	47.3 ± 5	5.17 ± 1	2.4 ± 0.5
	1100	44.08	4.4736		19.38 ± 0.03	34.7 ± 2	7.75	172.6 ± 12	24.09 ± 2	26.4 ± 3
	1200	40.32	4.7744		28.66 ± 0.03	52.70 ± 5	11.05	520 ± 10	36.4 ± 2	116.7 ± 8
SS316L + 30 vol % PMMA (Present study)	1000	61.18	3.1056	28 ± 2	1.99 ± 0.02	Very fragile parts failed at low compressive loading				
	1100	60.20	3.1840		3.81 ± 0.02	12.6 ± 2	3.95	34.9 ± 3	13.29 ± 3	3.9 ± 0.5
	1200	57.67	3.3864		4.13 ± 0.03	16.2 ± 3	4.78	75.4 ± 5	27.3 ± 2	13.8 ± 1
Femoral head [55]	–	–	–	–	2.9	68	–	–	–	–
Femoral condyle [55]	–	–	–	–	4.9	32	–	–	–	–
Vertebra [55]	–	–	–	–	1.5	4.1	–	–	–	–
SS316L ^1^ [19]	1100	57	–	160	1.58	15.5	4.50	–	–	–
SS316L ^1^ [19]	1200	45	–	–	3.27	36.3	8.25	–	–	–
SS316L ^1^ [19]	1300	30	–	35	6.64	52.8	9.42	–	–	–
SS316L ^1^ [51]	1150	46	–	–	–	25	5.78	–	–	–
SS316L ^2^ [12]	1200	71.5	–	1750–2350	1.05	14.1	6.18	–	–	–
SS316L ^2^ [12]	1200	64.8	–		2.03	28.8	10.22	–	–	–
SS316L ^3^ [56]	920 (103 MPa)	25.4	–	10–57	18	110	16.77	–	25	–
Austenitic Steel foam ^4^ [15]	1200	68	2.56	350–400	7.31	10.5	4.101	–	–	3.5 ± 0.25

* Note: ^1^ Selective laser sintering; ^2^ Powder metallurgy using carbamide as space holder; ^3^ Cold isostatic press followed by hot isostatic press, ^4^ Impregnation method.
